# *Saccharomyces boulardii* for the Prevention of Antibiotic-Associated Diarrhea in Hospitalized Patients Receiving Antibiotics: A Single-Center, Randomized, Double-Blind, Placebo-Controlled Trial

**DOI:** 10.3390/antibiotics15090868

**Published:** 2026-09-06

**Authors:** Pedja Kovacevic, Vladimir Mrdja, Tijana Kovacevic, Jelena Mrdja, Ivan Soldatovic, Zvezdana Rajkovaca

**Affiliations:** 1 Medical Intensive Care Unit, University Clinical Centre of the Republic of Srpska, Dvanaest beba bb, 78000 Banja Luka, Bosnia and Herzegovina; 2 Faculty of Medicine, University of Banja Luka, Save Mrkalja 14, 78000 Banja Luka, Bosnia and Herzegovina; tijana.kovacevic@kc-bl.com (T.K.); jelena.mrdja@kc-bl.com (J.M.); zvezdana.rajkovaca@kc-bl.com (Z.R.); 3 Department of Anesthesiology and Perioperative Critical Care, University Clinical Centre of the Republic of Srpska, 78000 Banja Luka, Bosnia and Herzegovina; vladimir.mrdja@kc-bl.com; 4 Faculty of Medicine, University of Belgrade, 11000 Belgrade, Serbia; ivan.soldatovic@med.bg.ac.rs

**Keywords:** *Saccharomyces boulardii*, antibiotic-associated diarrhea, probiotics, hospitalized adults

## Abstract

**Introduction:** Antibiotic-associated diarrhea (AAD) is a common complication of antibiotic therapy, while evidence supporting *Saccharomyces boulardii* for its prevention among hospitalized adults in resource-constrained settings remains limited. We evaluated the efficacy of Bularen^®^ in preventing AAD among hospitalized adults receiving systemic antibiotics. **Methods:** This single-center, randomized, double-blind, placebo-controlled trial was conducted at a tertiary care hospital in Bosnia and Herzegovina. Participants were randomly assigned to Bularen^®^ (*S. boulardii*) 250 mg twice daily or a matching placebo for 28 days. The primary outcome was at least one episode of AAD, defined as two or more loose or liquid stools within 24 h, during the 28-day follow-up. **Results:** Of 435 patients screened, 100 were randomized to Bularen^®^ (*n* = 51) or placebo (*n* = 49), and all completed follow-up. AAD occurred in 4/51 participants (7.8%) receiving Bularen^®^ and 35/49 (71.4%) receiving placebo (risk ratio, 0.11; 95% confidence interval, 0.04–0.29; *p* < 0.001), corresponding to an absolute risk reduction of 63.6 percentage points. During intravenous antibiotic therapy, AAD occurred in 0% versus 26.5% of participants, respectively (*p* < 0.001), and during oral therapy in 7.8% versus 51.0% (risk ratio, 0.15; 95% confidence interval, 0.06–0.41; *p* < 0.001). No toxin-positive *Clostridioides difficile* infections were identified. **Conclusions:** Bularen^®^ substantially reduced AAD incidence in this study population. Given the high placebo event rate and single-center design, confirmation in larger multicenter trials using standardized AAD definitions is warranted.

## 1. Introduction

Antibiotic-associated diarrhea (AAD) is defined as otherwise unexplained diarrhea occurring during or following antibiotic therapy and has been reported in up to 30% of patients receiving antibiotics [1,2,3]. Its clinical spectrum ranges from mild, self-limiting diarrhea to severe, potentially life-threatening disease. *Clostridioides difficile* infection (CDI) accounts for approximately 10–25% of AAD cases, and older hospitalized adults represent a particularly high-risk population [1,4]. AAD, particularly when caused by CDI, is associated with increased morbidity and mortality, prolonged hospitalization, modification or discontinuation of the inciting antibiotic therapy, severe complications including colitis, and increased healthcare costs [5,6]. Probiotics, including bacterial preparations and yeasts, are widely used for various clinical indications, with the prevention of AAD being among the most common. Antibiotic-induced disruption of the gut microbiota is considered a key mechanism underlying the development of AAD [7]. Accordingly, the co-administration of probiotics with antibiotics may help restore microbial balance, modulate immune responses, lower colonic pH to promote the growth of beneficial bacteria, and inhibit pathogens and their toxins [8].

Probiotics are live microorganisms that, when administered in adequate amounts, confer a health benefit on the host. Initially introduced in 2001 by a joint expert panel of the Food and Agriculture Organization of the United Nations (FAO) and the World Health Organization (WHO), this definition was reaffirmed by the International Scientific Association for Probiotics and Prebiotics (ISAPP) in 2014 [9,10]. Probiotics are available as dietary supplements, pharmaceutical preparations, and food products. To meet this definition, microorganisms must be viable when administered and provided in sufficient quantities to produce a beneficial effect.

The nonpathogenic yeast *Saccharomyces cerevisiae* var. *boulardii* (*S. boulardii*) is of particular interest because, unlike bacterial probiotics, it is not directly affected by concurrently administered antibacterial agents. Although generally considered safe, *S. boulardii* has rarely been associated with fungemia, primarily in critically ill or immunocompromised patients and those with central venous catheters [11,12]. Available studies suggest that *S. boulardii* may reduce the risk of AAD; however, substantial heterogeneity, small sample sizes, and a high risk of bias limit the certainty of the available evidence [13,14]. Evidence specifically addressing the efficacy of *S. boulardii* for the prevention of AAD among hospitalized adults in low-resource settings (LRS) remains limited [13]. Importantly, LRS should not be defined solely according to national income classification, because clinically relevant resource limitations may vary substantially between regions, institutions, and levels of care within the same country [15]. In the context of AAD and CDI, these limitations may include limited diagnostic and microbiological capacity, incomplete infection surveillance, limited antimicrobial stewardship and infection-prevention resources, and reduced access to affordable preventive interventions. Such constraints may contribute to the underrecognition of AAD and CDI, leaving their true burden uncertain. Given the clinical importance of AAD and CDI and the need for accessible and affordable preventive strategies in LRS, we evaluated the efficacy of Bularen^®^ 250 mg capsules in preventing AAD among hospitalized adults receiving systemic antibiotic therapy at a university-affiliated tertiary care hospital in Bosnia and Herzegovina. We hypothesized that Bularen^®^ would reduce the incidence of AAD during the 28-day follow-up period compared with placebo.

## 2. Materials and Methods

### 2.1. Study Design and Setting

This six-month, single-center, randomized, double-blind, placebo-controlled trial was conducted between December 2025 and May 2026 among hospitalized adults receiving systemic antibiotic therapy. The study was performed at the University Clinical Centre of the Republic of Srpska, a university-affiliated tertiary care hospital in Banja Luka, Bosnia and Herzegovina, which serves as the principal referral center for patients from across the Republic of Srpska. Participants were recruited from the Departments of Pulmonology, General and Abdominal Surgery, and Thoracic Surgery. These departments were selected to ensure the inclusion of both medical and surgical patients receiving systemic antibiotics, thereby enhancing the clinical representativeness of the study population. The study protocol was approved by the Ethics Committee of the University Clinical Centre of the Republic of Srpska (approval No. 01-19-236-2/25), and the trial was conducted in accordance with the principles of the Declaration of Helsinki. Written informed consent was obtained from all participants before enrollment. The trial was not prospectively registered in a publicly accessible clinical trial registry and is reported in accordance with the CONSORT 2010 Statement; the completed checklist is provided as Supplementary Material S1.

### 2.2. Participants

Participants were consecutively recruited from patients admitted to the participating departments. Eligibility criteria are presented in Table 1. All eligible patients received written information about the study objectives, procedures, and potential risks. Those who agreed to participate provided written informed consent before enrollment.

### 2.3. Study Intervention and Follow-Up

Potentially eligible patients were assessed according to the predefined inclusion and exclusion criteria, and written informed consent was obtained before enrollment. Baseline demographic and clinical characteristics, relevant risk factors, and available laboratory findings were then recorded, followed by randomization. Recruitment was continuous and included weekends. Participants in the intervention group received one Bularen^®^ 250 mg capsule (Abela Pharm, Serbia; distributed by Abela BiH, Bosnia and Herzegovina) twice daily under fasting conditions, at least two hours after a meal. Each capsule contained a lyophilized preparation of *Saccharomyces boulardii* providing at least 5 × 10^9^ viable colony-forming units (CFU), as specified by the manufacturer. Participants in the control group received placebo capsules manufactured by Abela Pharm and administered according to the same dosing schedule. The placebo capsules contained microcrystalline cellulose, maltodextrin, cocoa powder, corn starch, and magnesium stearate and were identical to Bularen^®^ capsules in appearance, taste, and odor, thereby maintaining participant blinding. The assigned intervention, *S. boulardii* or placebo, was initiated within 48 h of starting systemic antibiotic therapy and continued for 28 days. Antibiotic treatment was limited to a maximum of 21 days; patients requiring a longer course were excluded from the study. During hospitalization, administration of the assigned study product, including any refused doses, was monitored and documented by the clinical staff. Participants recorded the frequency and consistency of each bowel movement in a study diary, which served as the prespecified source document. Episodes of diarrhea were documented by the nursing staff and confirmed by a study investigator. When diarrhea occurred, a stool sample was collected. Stool samples were tested for *C. difficile* toxins A and B using the Premier^®^ Toxins A&B qualitative enzyme immunoassay (catalog No. 616096; Meridian Bioscience, Cincinnati, OH, USA). According to the manufacturer, the assay has a sensitivity of 94.7% (95% CI, 88.1–98.3%) and a specificity of 97.3% relative to the cellular cytotoxicity assay. At discharge, participants received sufficient capsules to complete the 28-day course and were instructed to contact the study team if diarrhea developed. Follow-up continued until day 28 after initiation of the assigned intervention. Outcome data and adherence were assessed through weekly telephone interviews and review of the completed study diaries. Adherence was additionally evaluated by counting unused capsules returned to the study team. Adverse events, including their clinical severity and any resulting discontinuation of the assigned study intervention, were documented throughout the 28-day follow-up.

### 2.4. Outcomes

Diarrhea was defined as the passage of two or more loose or liquid stools within 24 h. This definition was prespecified in the study protocol and applied identically to both randomized groups. Stool consistency was recorded descriptively as loose or liquid but was not classified using the Bristol Stool Form Scale, as this instrument was not included in the prespecified data-collection protocol. AAD was defined as otherwise unexplained diarrhea occurring after the initiation of systemic antibiotic therapy and within 28 days after initiation of the assigned study intervention. CDI was defined as AAD accompanied by a positive stool test for *C. difficile* toxins A and/or B.

The primary outcome was the occurrence of at least one episode of AAD during the 28-day follow-up period. Secondary outcomes included the occurrence of AAD during intravenous and oral antibiotic therapy, the daily number of loose or liquid stools among participants who developed AAD, the occurrence of CDI, and the occurrence of bloating during follow-up.

### 2.5. Randomization, Allocation Concealment, and Masking

Participants were randomly assigned in a 1:1 ratio to receive either *S. boulardii* or placebo using a computer-generated randomization sequence prepared by an independent statistician. Allocation concealment was maintained through sequentially numbered study-product containers prepared and dispensed by the hospital pharmacy. The containers were identical in appearance and labeled only with the corresponding participant identification code, without indicating treatment allocation. Bularen^®^ and placebo capsules were identical in appearance, taste, and odor. Participants, treating physicians, nurses, study investigators, microbiology personnel, and data analysts remained unaware of treatment assignments until the database was locked and the prespecified analyses were completed.

### 2.6. Sample Size

No formal a priori sample-size or statistical power calculation was performed. The study was designed as an exploratory, single-center randomized trial, and the target sample size of 100 participants was determined pragmatically based on recruitment feasibility during the predefined six-month study period. Accordingly, the estimates of treatment effect, particularly for secondary outcomes, should be interpreted as exploratory and require confirmation in an adequately powered multicenter trial.

### 2.7. Statistical Analysis

Statistical analyses were performed according to the intention-to-treat principle, with all randomized participants analyzed in the groups to which they were originally assigned. Continuous variables are presented as means with standard deviations when normally distributed and as medians with interquartile ranges when non-normally distributed. Normally distributed continuous variables were compared using the independent-samples *t* test, whereas non-normally distributed variables were compared using the Mann–Whitney *U* test. Categorical variables are presented as numbers and percentages and were compared using Pearson’s chi-square test or Fisher’s exact test, as appropriate. The Fisher–Freeman–Halton exact test was used for categorical variables with more than two categories when expected cell counts were small. The primary outcome, defined as the occurrence of at least one episode of AAD during the 28-day follow-up period, was compared between the Bularen^®^ and placebo groups using Pearson’s chi-square test. To assess whether the association between treatment allocation and the overall occurrence of AAD was independent of the baseline sex imbalance and other potentially relevant covariates, a post hoc multivariable binary logistic regression analysis was performed. The occurrence of AAD during the overall 28-day follow-up period was entered as the dependent variable. Treatment allocation (*S. boulardii* vs. placebo), sex, age, surgical status, intravenous cephalosporin exposure, meropenem exposure, and cefixime exposure were entered simultaneously as independent variables. Age was analyzed as a continuous variable, whereas all other variables were treated as binary categorical variables. The results are presented as adjusted odds ratios (aORs) with 95% confidence intervals (CIs). The overall significance of the model was assessed using the likelihood-ratio chi-square test. Secondary binary outcomes, including AAD during intravenous and oral antibiotic therapy and the occurrence of bloating, were analyzed using Pearson’s chi-square test or Fisher’s exact test, as appropriate. Between-group differences in the number of loose or liquid stools were assessed using the Fisher–Freeman–Halton exact test. Secondary outcome analyses were considered exploratory, and no adjustment for multiple comparisons was applied. All statistical tests were two-sided, and a *p* value < 0.05 was considered statistically significant. Analyses were performed using IBM SPSS Statistics, version 31 (IBM Corp., Armonk, NY, USA).

## 3. Results

**Figure 1:**
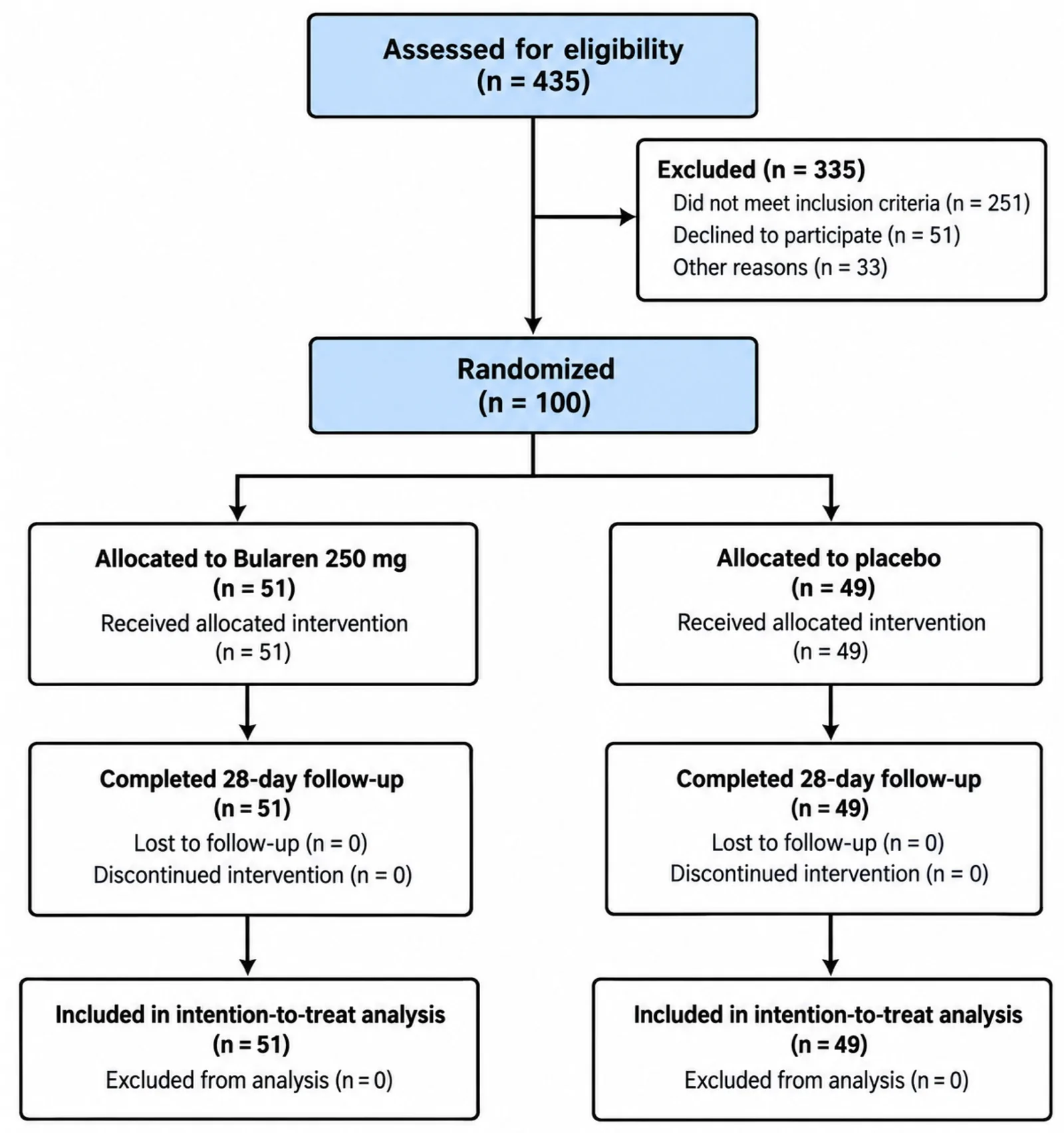
Flow of patients through screening, randomization, follow-up, and intention-to-treat analysis.

Among the 435 patients assessed for participation, 335 were not enrolled. The reasons for non-enrollment were failure to satisfy the eligibility criteria in 251 cases, refusal to participate in 51 cases, and other reasons in 33 cases. The trial population therefore comprised 100 randomized participants, of whom 51 received Bularen^®^, and 49 received placebo (Figure 1). Follow-up through day 28 was completed for every randomized participant, and no participant was excluded from the intention-to-treat analysis. Primary-outcome data were consequently available for the entire trial population. Study diaries and counts of returned capsules indicated complete adherence to the allocated intervention in both groups.

Participants allocated to Bularen^®^ had a mean age of 60.2 ± 14.3 years, compared with 59.9 ± 17.3 years among those allocated to placebo. Men represented 21 of 51 participants (41.2%) in the Bularen^®^ group and 30 of 49 participants (61.2%) in the placebo group. Apart from this difference in sex distribution, the recorded demographic and treatment characteristics were comparable between the groups (Table 2). Combined intravenous and oral antibiotic treatment was administered to 46 participants (90.2%) receiving Bularen^®^ and 43 participants (87.8%) receiving placebo. The remaining 5 participants (9.8%) in the Bularen^®^ group and 6 participants (12.2%) in the placebo group received intravenous antibiotics only. In each group, the median number of intravenous antibiotics was 2 (IQR, 1–2; *p* = 0.944), whereas the median number of orally administered antibiotics was 1 (IQR, 1–1; *p* = 0.944). Intravenous treatment lasted a median of 6 days (IQR, 4–8) in the Bularen^®^ group and 7 days (IQR, 3–10) in the placebo group (*p* = 0.526). The median duration of oral treatment was 7 days (IQR, 5–7) in both groups (*p* = 0.609). The proportions of participants managed operatively and nonoperatively were also comparable (*p* = 0.543). No statistically significant difference was identified between the groups in exposure to any individual antibiotic agent evaluated (all *p* ≥ 0.060). A complete account of the antibiotics administered and their distribution by treatment group is available in the Supplementary Material S2.

### 3.1. Primary Outcome

During the 28-day observation period, at least one episode of antibiotic-associated diarrhea was recorded in 4 of the 51 participants receiving Bularen^®^ (7.8%) and in 35 of the 49 participants receiving placebo (71.4%). This corresponded to a risk ratio of 0.11 (95% CI, 0.04–0.29; *p* < 0.001). The absolute between-group difference in risk was 63.6 percentage points in favor of Bularen^®^ (Table 3).

### 3.2. Secondary Outcomes

The occurrence of AAD was also examined separately according to the route of ongoing antibiotic treatment. During intravenous therapy, none of the 51 participants allocated to Bularen^®^ experienced AAD, whereas the outcome was documented in 13 of the 49 placebo recipients (26.5%; *p* < 0.001). Each of these 13 episodes involved two loose or liquid stools within a 24 h period.

While participants were receiving oral antibiotics, AAD was identified in 4 of 51 participants in the Bularen^®^ group (7.8%) and 25 of 49 participants in the placebo group (51.0%). The resulting risk ratio was 0.15 (95% CI, 0.06–0.41; *p* < 0.001). All four affected participants receiving Bularen^®^ reported two loose or liquid stools within 24 h. Of the 25 affected placebo recipients, 21 (84.0%) reported two such stools, and 4 (16.0%) reported three. The groups did not differ in the distribution of daily stool frequency among participants who developed AAD during oral antibiotic treatment (*p* = 1.000).

Stool specimens were collected from all 39 participants in whom AAD was documented. Tests for *Clostridioides difficile* toxins A and B were negative in every specimen using the employed enzyme immunoassay; therefore, no participant met the prespecified study definition of toxin-positive CDI. Bloating was reported by 1 of 51 participants receiving Bularen^®^ (2.0%) and 37 of 49 participants receiving placebo (75.5%; *p* < 0.001). The only other reported gastrointestinal adverse event was mild intestinal cramping. No moderate or severe adverse events, adverse-event-related treatment discontinuations, or serious adverse events occurred in either randomized group. The secondary-outcome findings are summarized in Table 4.

To account for the baseline sex imbalance and other potentially relevant covariates, a post hoc multivariable binary logistic regression analysis was performed. After simultaneous adjustment for sex, age, surgical status, intravenous cephalosporin exposure, meropenem exposure, and cefixime exposure, treatment with *S. boulardii* remained independently associated with substantially lower odds of AAD (aOR 0.014, 95% CI 0.003–0.070; *p* < 0.001). Sex, age, surgical status, and the included antibiotic exposures were not independently associated with AAD (Table 5). The overall model was statistically significant (likelihood-ratio χ^2^(7) = 54.51; *p* < 0.001).

## 4. Discussion

The principal finding of this randomized trial was a markedly lower occurrence of antibiotic-associated diarrhea among hospitalized adults allocated to Bularen^®^ than among those receiving placebo. Over the 28-day observation period, AAD was documented in 7.8% of participants in the Bularen^®^ group and 71.4% of participants in the placebo group, yielding a risk ratio of 0.11 and an absolute difference of 63.6 percentage points. The direction of the treatment effect remained unchanged when AAD was assessed separately during intravenous and oral antibiotic administration. These phase-specific findings support the primary result, but they should not be viewed as independent confirmatory evidence because they were secondary analyses and no adjustment was made for multiple comparisons. Furthermore, all stool specimens obtained during AAD episodes were negative for *Clostridioides difficile* toxins A and/or B. Accordingly, this study primarily evaluates the prevention of AAD in participants in whom *C. difficile* toxins were not detected by the employed enzyme immunoassay and does not provide evidence that Bularen^®^ prevents CDI.

Previous randomized studies of *S. boulardii* have produced considerably different estimates of its effect on AAD. In the systematic review by Szajewska and Kołodziej, which included 21 randomized trials and 4780 participants, AAD occurred in 8.5% of participants receiving *S. boulardii* and 18.7% of controls, corresponding to a pooled risk ratio of 0.47 (95% CI, 0.38–0.57) [13]. Although these pooled findings favored *S. boulardii*, results from individual trials in hospitalized adults have not been uniform. Pozzoni et al. reported AAD in 15.1% of participants treated with *S. boulardii* and 13.3% of those receiving placebo, with no statistically significant treatment advantage [16]. Similarly, the multicenter trial conducted by Ehrhardt et al. recorded 21 AAD events in the *S. boulardii* group and 19 in the placebo group and found no evidence of a preventive effect [14]. Thus, the direction of our primary result is compatible with the pooled evidence, but the magnitude of the difference observed in our trial is substantially greater than that reported in most previous adult studies.

The unusually high incidence of AAD in our placebo group is central to the interpretation of the effect estimate. Several aspects of the study may have increased the detection of bowel changes that would not have met the outcome definition in trials using more restrictive criteria. We defined diarrhea as two or more loose or liquid stools within 24 h, whereas other studies have frequently required at least three unformed stools per day, persistence beyond a specified period, classification according to the Bristol Stool Form Scale, or additional clinical criteria [2,5,13,14,16]. The Bristol Stool Form Scale was not incorporated into the prespecified data-collection protocol, and stool consistency was therefore documented descriptively rather than assigned a formal Bristol type. Although our definition was prespecified and applied identically to both randomized groups, its high sensitivity may have captured mild or transient changes in bowel habits that would not have qualified as AAD under more restrictive definitions. In addition, participants had substantial cumulative antibiotic exposure. Nearly 90% received sequential intravenous and oral antibiotic treatment, with a median exposure to two intravenous antibiotics followed by one oral antibiotic. The number and duration of antibiotic exposures are recognized determinants of AAD risk [2,5], plausibly through greater disruption of the intestinal microbiota. Therefore, cumulative antibiotic exposure in our study population may have increased baseline susceptibility to antibiotic-associated changes in bowel habits. Bowel movements were recorded prospectively in study diaries, events during hospitalization were verified by the clinical staff, and follow-up continued after discharge until day 28. This active surveillance strategy may have captured episodes that would have been missed by retrospective assessment or monitoring restricted to the hospitalization period. The recorded events were predominantly characterized by two loose or liquid stools within 24 h, and a substantial proportion would therefore not have met more restrictive AAD definitions. Because the duration of individual diarrhea episodes was not recorded, a post hoc sensitivity analysis applying a definition of at least three loose or liquid stools per day for two or more consecutive days could not be performed. Consequently, the robustness of the observed treatment effect under this more restrictive definition cannot be established from the available data. Although the prespecified definition was applied identically to both groups, its high sensitivity probably increased the absolute event rates and may have magnified the apparent relative and absolute treatment effects. Accordingly, the observed risk ratio and absolute risk reduction should be regarded as definition-dependent estimates and should not be compared directly with results from trials using more restrictive or duration-based definitions. The magnitude of the observed effect should therefore be considered exploratory until confirmed in an adequately powered trial using a harmonized AAD definition.

Heterogeneity among probiotic trials may also arise from differences in the enrolled populations and interventions. Baseline susceptibility to AAD is influenced by age, comorbidities, hospitalization, the number and classes of antibiotics administered, duration of exposure, and local patterns of clinical and microbiological practice [1,2,5]. Studies have also evaluated different *S. boulardii* products, viable organism counts, treatment schedules, timing of administration relative to antibiotic initiation, and follow-up periods [13,14,16,17]. A preventive intervention may appear to have a larger absolute effect when tested in a population with a high underlying risk of AAD, even if its biological effect is comparable to that observed elsewhere. Conversely, trials enrolling populations at relatively low baseline risk may have insufficient outcome events to demonstrate a difference. Direct numerical comparisons between our trial and previous studies should therefore be made cautiously, particularly because the case definitions and surveillance procedures were not identical.

The absence of AAD during intravenous antibiotic therapy in the Bularen^®^ group, compared with 13 cases in the placebo group, suggests that the between-group difference developed early during antibiotic exposure. A lower AAD frequency was also observed during oral treatment, when events occurred in 7.8% and 51.0% of participants in the Bularen^®^ and placebo groups, respectively. Although the similarity in the direction of these findings is reassuring, the intravenous and oral treatment periods should not be interpreted as fully independent exposure phases. Most participants received antibiotics by both routes, and an individual participant could contribute clinical information across successive phases of treatment. These analyses were also exploratory and were not supported by a multiplicity-adjusted statistical plan. Their appropriate role is therefore to describe the timing and clinical pattern of the primary outcome rather than to establish route-specific efficacy.

Among participants who developed AAD during oral antibiotic treatment, the recorded number of loose or liquid stools per day did not differ significantly between the treatment groups. All four affected participants receiving Bularen^®^ had two such stools within 24 h, compared with two stools in 21 placebo recipients and three stools in four placebo recipients. This subgroup was small, particularly in the intervention arm, and the study was not designed to determine whether *S. boulardii* modifies the intensity or duration of diarrhea once an episode has developed. Ehrhardt et al. similarly found no demonstrable effect on stool frequency or diarrhea duration [14], while the wider adult literature remains inconsistent regarding clinical effects beyond prevention of the first AAD episode [17]. The present findings, therefore, suggest a difference in the occurrence of AAD but should not be interpreted as evidence that Bularen^®^ reduces episode severity.

No toxin-positive CDI was identified among the 39 participants who developed AAD. However, the reported sensitivity of the employed enzyme immunoassay is 94.7%, and false-negative results cannot be excluded. Because no confirmatory nucleic acid amplification test or toxigenic culture was performed, some CDI cases may have remained undetected. Our findings should therefore be interpreted as showing that no toxin-positive CDI was identified using the employed assay rather than as definitively excluding CDI in all participants with AAD. Consequently, the absence of detected toxin-positive CDI events prevents any estimation of treatment efficacy for this outcome. Earlier adult trials also recorded few CDI cases, thereby limiting their statistical power to evaluate CDI prevention [14,16]. The meta-analysis by Szajewska and Kołodziej did not demonstrate a significant reduction in CDI among adults, although a benefit was reported in children [13]. Other systematic reviews have likewise found insufficient or inconsistent evidence for the use of *S. boulardii* in primary CDI prevention, while suggesting that selected probiotic regimens may have a role in reducing recurrence in particular patient populations [18,19]. Primary prevention of AAD and prevention of CDI should therefore remain conceptually separate outcomes. Our results support neither a favorable nor an unfavorable conclusion regarding CDI.

Bloating was reported by 2.0% of participants receiving Bularen^®^ and 75.5% of those receiving the placebo. This marked difference is potentially clinically relevant but requires especially cautious interpretation. Bloating was a secondary, subjectively reported outcome; it was not graded according to severity and was analyzed without correction for multiple comparisons. In addition, the trial was not primarily designed or powered to evaluate bloating. Previous research has suggested that *S. boulardii* may reduce gastrointestinal symptoms associated with antibiotic-containing treatment regimens, including abdominal distension; however, much of that evidence originates from children or from patients receiving multidrug therapy for *Helicobacter pylori* eradication [20]. Those populations and treatment contexts differ substantially from the hospitalized adults included in our trial. The present bloating result should, therefore, be regarded as hypothesis-generating and should be assessed using validated symptom measures in future studies.

Evidence from adult inpatient populations outside high-income Western healthcare systems remains relatively limited. A prospective study in hospitalized adults in Turkey reported fewer cases of AAD among patients receiving prophylactic *S. boulardii*, whereas a Chilean trial involving adult outpatients treated with amoxicillin did not identify a significant preventive effect [21,22,23]. A more recent meta-analysis of 46 randomized trials conducted in China also favored *S. boulardii* for AAD prevention, but approximately 96% of the included studies involved children, open control groups were frequently used, and evidence of publication bias was identified [21]. These characteristics restrict direct comparison with a double-blind, placebo-controlled trial in hospitalized adults. Our study contributes evidence from a tertiary hospital in Southeast Europe, a region that has been underrepresented in previous research on probiotic prevention of AAD.

Nevertheless, the study location should not by itself be used to assume that the results apply to all low-resource settings. National income categories provide only a broad socioeconomic description and do not capture differences in diagnostic capacity, staffing, antimicrobial stewardship, infection surveillance, medication availability, or access to preventive interventions across individual healthcare facilities [15]. Resource limitations may coexist with advanced services within the same country or institution, and clinically important constraints can also occur in nominally higher-income environments. The external validity of our findings should therefore be assessed according to the characteristics of the participating hospital, its patients, patterns of antibiotic use, and the methods available for recognizing AAD and CDI. Confirmation in institutions with different resource profiles would be more informative than extrapolation based solely on national or regional classification.

Several design features strengthen the internal interpretation of this trial. Treatment allocation was generated independently and concealed using sequentially numbered study-product containers. The active preparation and placebo were matched in appearance, taste, and odor, and participants, clinical personnel, investigators, microbiology staff, and data analysts remained unaware of treatment assignment until completion of the prespecified analyses. Outcome surveillance was prospective, primary-outcome information was available for all randomized participants, and follow-up was completed through day 28. The intention-to-treat analysis consequently included the complete randomized population. These procedures reduce the likelihood that differential follow-up, knowledge of treatment allocation, or post-randomization exclusion explains the observed difference.

Importantly, the association between *S. boulardii* treatment and lower odds of antibiotic-associated diarrhea remained robust in the post hoc multivariable analysis. Adjustment for the baseline sex imbalance, age, surgical status, and relevant antibiotic exposures did not attenuate the observed treatment effect. None of these covariates were independently associated with AAD, suggesting that the substantial between-group difference was unlikely to be explained by the observed sex imbalance or by differences in the included clinical and antibiotic-related characteristics. Nevertheless, because the adjusted analysis was post hoc and the sample size was limited, the magnitude of the adjusted association should be interpreted cautiously and confirmed in larger multicenter trials with prespecified covariate-adjusted analyses.

Important uncertainties, nevertheless, remain. First, no formal a priori sample-size or statistical power calculation was performed, and the target sample size of 100 participants was determined pragmatically based on recruitment feasibility during the predefined six-month study period. The modest sample size and single-center design limit the precision of the treatment-effect estimate, particularly for secondary and subgroup analyses, and restrict the range of patients and clinical practices represented in the study. The magnitude of the observed effect should therefore be regarded as exploratory and requires confirmation in an adequately powered multicenter randomized trial. Second, the prespecified definition of diarrhea was more sensitive than definitions commonly applied in other AAD trials and probably resulted in the inclusion of mild or transient bowel disturbances that would not have met more restrictive criteria. Because the recorded events were predominantly characterized by two loose or liquid stools within 24 h, the sensitive definition may have increased the absolute event rates and magnified the apparent relative and absolute treatment effects. Stool consistency was not formally classified using the Bristol Stool Form Scale. Consequently, the observed effect estimate is definition-dependent and cannot be compared directly with estimates from trials using more restrictive or standardized AAD definitions. Third, an imbalance in sex distribution occurred after randomization. Although the association between treatment allocation and AAD remained significant after adjustment for sex, age, surgical status, and selected antibiotic exposures, randomization in a modest sample cannot ensure balance in every measured or unmeasured determinant of AAD. Individual-level comorbidity data were not available for inclusion in the adjusted analysis. Therefore, residual confounding related to comorbidities, diet, gastrointestinal susceptibility, antibiotic exposures not included in the model, or other unmeasured factors cannot be completely excluded.

Additional limitations affect the interpretation of the secondary outcomes and the applicability of the results. The analyses according to intravenous and oral antibiotic treatment were exploratory and were not adjusted for multiple comparisons. Bloating was based on participant reporting and was not assessed using a validated scale or predefined categories of severity. The absence of detected toxin-positive CDI cases, together with the possibility of false-negative enzyme immunoassay results, made evaluation of CDI prevention impossible. Patients with immunodeficiency, active malignancy, central venous catheters, or enteral feeding were excluded, reducing the applicability of the findings to populations in whom probiotic safety may be of particular concern. The sample was also too small to assess rare adverse events. Finally, the results relate specifically to Bularen^®^ administered at the evaluated dose and for the specified 28-day period. They should not be generalized automatically to *S. boulardii* preparations with different manufacturing characteristics, viable organism counts, dosing schedules, or durations of administration.

In summary, Bularen^®^ administration was associated with a substantially lower 28-day incidence of AAD than placebo among the hospitalized adults enrolled in this trial. A similar direction of effect was observed during the intravenous and oral phases of antibiotic treatment, while no conclusion could be reached regarding CDI prevention. The size of the observed treatment difference should be interpreted in the context of the unexpectedly high placebo-group event rate, the sensitive two-stool definition of diarrhea, and the limited single-center sample. Replication in adequately powered multicenter trials using harmonized AAD definitions, standardized assessment of gastrointestinal symptoms, and enrollment across healthcare institutions with different patient and resource profiles is necessary before the effect estimate can be considered broadly generalizable.

## Figures and Tables

**Table 1:** Eligibility criteria for study participation.

**Inclusion Criteria:**
Adult patient aged ≥18 years.
Hospitalization in one of the participating departments.
Treatment with at least one systemic antibiotic.
Legal capacity to provide informed consent.
Ability to understand and comply with the study procedures.
Provision of written informed consent before enrollment.
**Exclusion Criteria:**
Age <18 years.
Known hypersensitivity to yeast or any component of Bularen^®^ 250 mg capsules.
Pregnancy or breastfeeding.
Gastrointestinal bleeding.
Primary or secondary immunodeficiency.
Active malignancy.
Systemic antibiotic therapy within six weeks before enrollment.
Regular use of products containing *S. boulardii* within seven days before enrollment.
Acute, ongoing, or chronic diarrhea at the time of enrollment.
Presence of a central venous catheter or anticipated need for central venous catheterization during the study period.
Enteral feeding.
Anticipated duration of systemic antibiotic therapy exceeding 21 days.

**Table 2:** Participant characteristics and antibiotic exposure by randomized treatment group.

	Bularen^®^ (*n* = 51)	Placebo (*n* = 49)	*p* Value
Male sex, *n* (%)	21 (41.2%)	30 (61.2%)	0.045 ^a^
Age, years	60.2 ± 14.3	59.9 ± 17.3	0.926 ^b^
**Route of antibiotic administration**			
Intravenous only	5 (9.8%)	6 (12.2%)	0.697 ^a^
Intravenous and oral	46 (90.2%)	43 (87.8%)	
Number of intravenous antibiotics	2 (1–2)	2 (1–2)	0.944 ^c^
Duration of intravenous antibiotic therapy, days	6 (4–8)	7 (3–10)	0.526 ^c^
Number of oral antibiotics	1 (1–1)	1 (1–1)	0.944 ^c^
Duration of oral antibiotic therapy, days	7 (5–7)	7 (5–7)	0.609 ^c^
**Treatment approach**			0.543 ^a^
Nonoperative	26 (51.0%)	22 (44.9%)	
Operative	25 (49.0%)	27 (55.1%)	

Data are expressed as mean ± standard deviation, median (interquartile range), or number (percentage), as appropriate. (a) Pearson’s chi-square test; (b) independent-samples t test; (c) Mann–Whitney U test.

**Table 3:** Occurrence of antibiotic-associated diarrhea within 28 days according to randomized treatment group.

Primary Outcome	Bularen^®^ (*n* = 51)	Placebo (*n* = 49)	Risk Ratio (95% CI)	*p* Value
At least one episode of AAD during the 28-day follow-up period	4 (7.8%)	35 (71.4%)	0.11 (0.04–0.29)	<0.001

Data are presented as number (percentage). AAD, antibiotic-associated diarrhea; CI, confidence interval.

**Table 4:** Secondary outcomes by randomized treatment group.

Outcome	Bularen^®^ (*n* = 51)	Placebo (*n* = 49)	*p* Value
Diarrhea during intravenous antibiotic therapy, *n* (%)	0 (0%)	13 (26.5%)	
2 stools/day	0 (0%)	13 (100.0%)	<0.001 ^a^
Diarrhea during oral antibiotic therapy, *n* (%)	4 (7.8%)	25 (51%)	<0.001 ^a^
2 stools/day	4 (100%)	21 (84%)	1.000 ^b^
3 stools/day	0 (0%)	4 (16%)	
Bloating	1 (2%)	37 (75.5%)	<0.001 ^a^
CDI, *n* (%)	0 (0.0%)	0 (0.0%)	-

Data are expressed as numbers (percentages). Percentages describing daily stool frequency were calculated only among participants who developed AAD during the corresponding phase of antibiotic treatment. AAD, antibiotic-associated diarrhea; CDI, Clostridioides difficile infection. (a) Pearson’s chi-square test; (b) Fisher’s exact test.

**Table 5:** Multivariable logistic regression analysis of factors associated with antibiotic-associated diarrhea.

Variable	Adjusted OR	95% CI	*p*-Value
*S. boulardii* vs. placebo	0.014	0.003–0.070	<0.001
Male sex	0.503	0.145–1.745	0.279
Age, per one-year increase	0.979	0.940–1.020	0.305
Surgical status	0.503	0.119–2.134	0.352
Intravenous cephalosporin exposure	0.951	0.274–3.295	0.937
Meropenem exposure	2.694	0.433–16.751	0.288
Cefixime exposure	0.203	0.041–1.018	0.053

## Data Availability

The original contributions presented in this study are included in the article/Supplementary Material. Further inquiries can be directed to the corresponding author.

## Supplementary Materials

The following supporting information can be downloaded at https://www.mdpi.com/article/10.3390/antibiotics15090868/s1. Supplementary Material S1: Completed CONSORT 2010 checklist; Supplementary Material S2: Complete account of the antibiotics administered and their distribution by randomized treatment group.
