# Supplementary material for: Saccharomyces boulardii for the Prevention of Antibiotic-Associated Diarrhea in Hospitalized Patients Receiving Antibiotics: A Single-Center, Randomized, Double-Blind, Placebo-Controlled Trial
Source: Antibiotics (Basel). 2026 Sep 6;15(9):868. doi: 10.3390/antibiotics15090868 (PMC13603640; doi:10.3390/antibiotics15090868)
Supplement: Supplementary file 1 [file antibiotics-15-00868-s001.zip › Supplementary Material S1.pdf]

CONSORT 2010 Checklist

Saccharomyces boulardii for the Prevention of Antibiotic-Associated Diarrhea in Hospitalized Patients Receiving Antibiotics: A Single-Center, Randomized, Double-Blind, Placebo-Controlled Trial

Section/topic	Item	Checklist item	Reported on page / location
**Title and abstract**	**1a**	Identify the study as a randomized trial in the title.	Page 1 (title)
	**1b**	Provide a structured summary of the trial design, methods, results, and conclusions.	Page 2 (Abstract)
**Introduction: background**	**2a**	Explain the scientific background and rationale.	Pages 2–3 (Introduction)
**Introduction: objectives**	**2b**	State the specific objective or hypothesis.	Page 3 (end of Introduction)
**Methods: trial design**	**3a**	Describe the trial design, including the allocation ratio.	Pages 3 and 5 (Sections 2.1 and 2.5)
	**3b**	Report important changes to methods after trial commencement and explain why they occurred.	Not applicable; no changes after trial commencement are reported
**Methods: participants**	**4a**	State participant eligibility criteria.	Pages 3–4 (Section 2.2; Table 1)
	**4b**	Describe the settings and locations where data were collected.	Page 3 (Section 2.1)
**Methods: interventions**	**5**	Describe each group's intervention in enough detail to allow replication, including how and when it was administered.	Pages 4–5 (Section 2.3)
**Methods: outcomes**	**6a**	Define all prespecified primary and secondary outcomes, including how and when they were assessed.	Page 5 (Section 2.4)
	**6b**	Report changes to trial outcomes after commencement and provide reasons.	Not applicable; no changes to prespecified outcomes are reported
**Methods: sample size**	**7a**	Explain how the sample size was determined.	Page 5 (Section 2.6; no formal a priori calculation)
	**7b**	When applicable, explain interim analyses and stopping rules.	Not applicable; no interim analysis or early-stopping rule
**Methods: sequence generation**	**8a**	Describe the method used to generate the random allocation sequence.	Page 5 (Section 2.5)
	**8b**	Describe the randomization type and any restrictions, such as blocking or block size.	Page 5 (Section 2.5; 1:1 allocation; no restrictions reported)
**Methods: allocation concealment**	**9**	Describe how the allocation sequence was concealed until participants were assigned.	Page 5 (Section 2.5)
**Methods: implementation**	**10**	State who generated the sequence, enrolled participants, and assigned interventions.	Page 5 (Section 2.5; independent statistician and hospital pharmacy reported)
**Methods: blinding**	**11a**	State who was blinded after assignment and how blinding was maintained.	Page 5 (Section 2.5)
	**11b**	When relevant, describe the similarity of the interventions.	Pages 4–5 (Sections 2.3 and 2.5)
**Methods: statistical analysis**	**12a**	Describe statistical methods used for primary and secondary outcomes.	Pages 5–6 (Section 2.7)
	**12b**	Describe methods for additional analyses, including subgroup or adjusted analyses.	Page 6 and pages 8–9 (exploratory phase-specific analyses)

Section/topic	Item	Checklist item	Reported on page / location
**Results: participant flow**	**13a**	For each group, report numbers randomized, treated as allocated, and included in the primary analysis.	Pages 6–7 (Results; Figure 1)
	**13b**	For each group, report losses and exclusions after randomization, with reasons.	Pages 6–7 (Figure 1; no losses or post-randomization exclusions)
**Results: recruitment**	**14a**	Report the dates defining recruitment and follow-up periods.	Page 3 (Section 2.1)
	**14b**	Explain why the trial ended or was stopped.	Page 5 (predefined six-month study period; no early termination)
**Results: baseline data**	**15**	Present baseline demographic and clinical characteristics by group.	Pages 7–8 (Table 2)
**Results: numbers analyzed**	**16**	For each analysis, report the number of participants included and whether groups were analyzed as randomized.	Page 6 and pages 8–9 (intention-to-treat analysis; Tables 3–4)
**Results: outcomes and estimation**	**17a**	For each outcome, report group results, effect size, and precision such as a 95% confidence interval.	Page 2 and pages 8–9 (Tables 3–4)
	**17b**	For binary outcomes, report both absolute and relative effect sizes.	Page 8 (Section 3.1; Table 3)
**Results: ancillary analyses**	**18**	Report other analyses performed, distinguishing prespecified from exploratory analyses.	Page 6 and pages 8–9 (Section 2.7; Section 3.2)
**Results: harms**	**19**	Report important harms or unintended effects in each group.	Pages 5 and 9 (adverse-event monitoring and results)
**Discussion: limitations**	**20**	Discuss limitations, potential sources of bias or imprecision, and multiplicity where relevant.	Pages 12–13 (Discussion)
**Discussion: generalizability**	**21**	Discuss the external validity and generalizability of the findings.	Pages 12–13 (Discussion)
**Discussion: interpretation**	**22**	Interpret results consistently with benefits, harms, limitations, multiplicity, and other evidence.	Pages 9–13 (Discussion)
**Other information: registration**	**23**	Provide the trial registry and registration number.	Pages 3 and 14: not prospectively registered; no registration number
**Other information: protocol**	**24**	State where the full trial protocol can be accessed, if available.	Not publicly available / not reported
**Other information: funding**	**25**	Report funding or other support and the role of funders.	Pages 13–14: no external funding; conflicts of interest declared absent

Note: Page numbers refer to the 15-page revised manuscript supplied for review. The checklist records reporting status transparently; it does not imply that unreported or non-applicable procedures were performed.
