# Supplementary material for: Saccharomyces boulardii for the Prevention of Antibiotic-Associated Diarrhea in Hospitalized Patients Receiving Antibiotics: A Single-Center, Randomized, Double-Blind, Placebo-Controlled Trial
Source: Antibiotics (Basel). 2026 Sep 6;15(9):868. doi: 10.3390/antibiotics15090868 (PMC13603640; doi:10.3390/antibiotics15090868)
Supplement: Supplementary file 1 [file antibiotics-15-00868-s001.zip › Supplementary Material S2.pdf]

SUPPLEMENTAL FILE

This Supplemental File has been provided by the authors for additional information about their work

Saccharomyces boulardii for the Prevention of Antibiotic-Associated Diarrhea in Hospitalized Patients Receiving Antibiotics: A Single-Center, Randomized, Double-Blind, Placebo-Controlled Trial

Pedja Kovacevic[1,2], Vladimir Mrdja[3], Tijana Kovacevic[1,4], Ivan Soldatovic[5], Jelena Mrdja[2], Zvezdana Rajkovaca[2]

1- Medical Intensive Care Unit, University Clinical Centre of the Republic of Srpska, 78000 Banja Luka, Bosnia and Herzegovina

2- Faculty of Medicine, University of Banja Luka, 78000 Banja Luka, Bosnia and Herzegovina

3- Department of Anesthesiology and Perioperative Critical Care, University Clinical Centre of the Republic of Srpska, 78000 Banja Luka, Bosnia and Herzegovina

4- Faculty of Medicine, University of Belgrade, 11000 Belgrade, Serbia.

∗ Corresponding author:

Pedja Kovacevic: e-mail: pedja.kovacevic@med.unibl.org, Dvanaest beba bb, Banja Luka, 78000, Bosnia and Herzegovina, tel. +38751342603; Fax: +38751342507

Groups

	N	%
Bularen®	51	51.0
Placebo	49	49.0
All	100	100.0

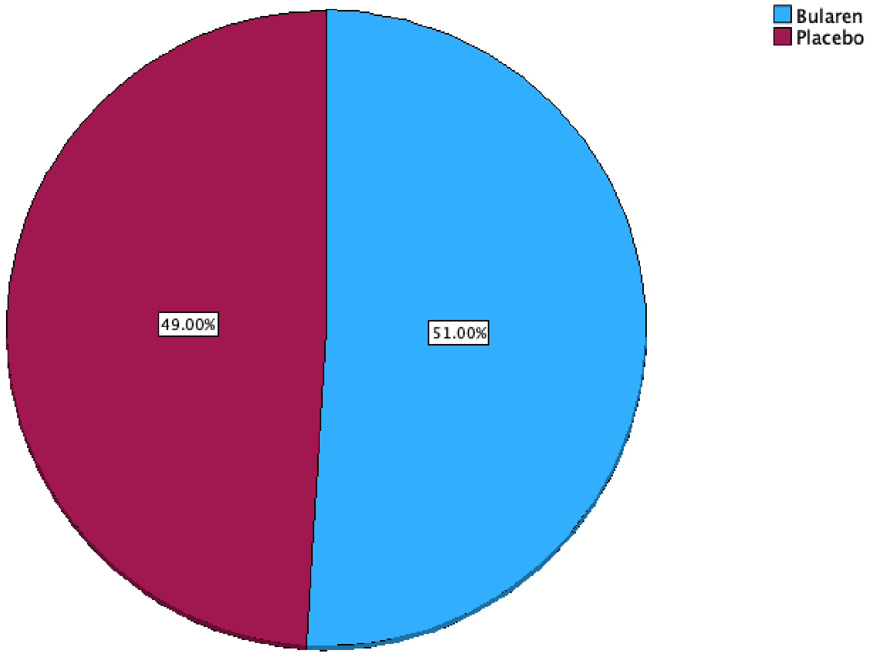

Age

		Treatment	
		Bularen®	Placebo
Age	N	51	49
	AS	60.2	59.9
	SD	14.3	17.3
	Min	32.0	18.0
	P25	46.0	48.0
	Med	63.0	65.0
	P75	71.0	70.0
	Max	88.0	85.0

t=0.094; p=0.926;

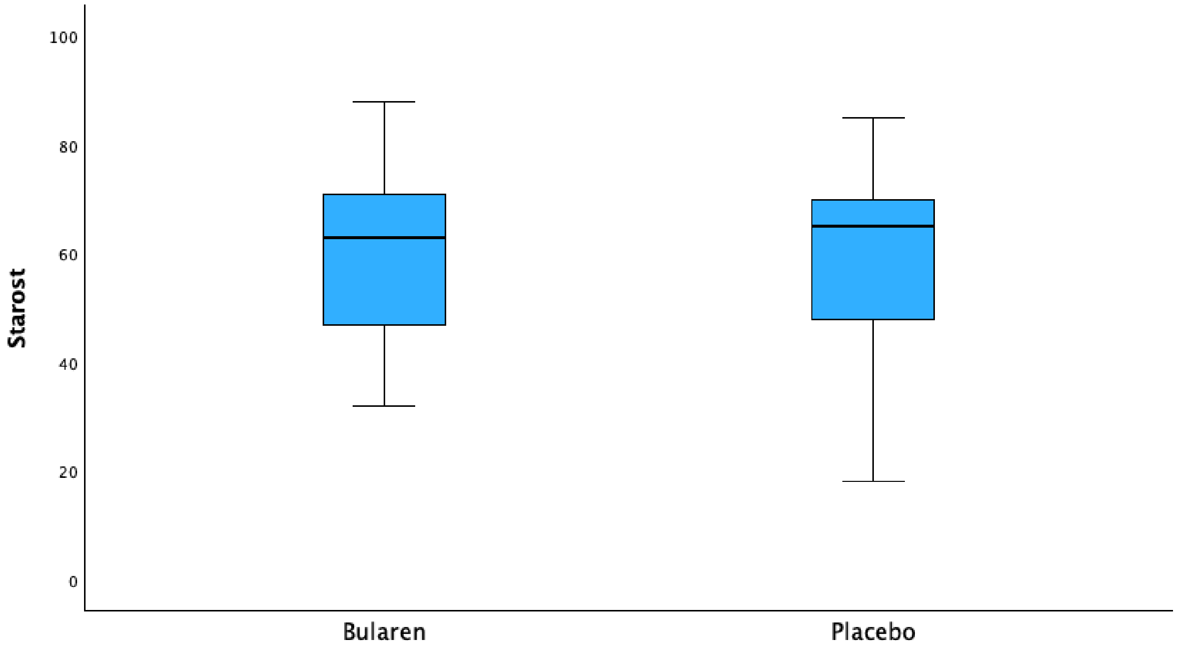

Sex

			Sex		
			Male	Female	All
Treatment	Bularen®	N	21	30	51
		%	41.2%	58.8%	100.0%
	Placebo	N	30	19	49
		%	61.2%	38.8%	100.0%
All		N	51	49	100
		%	51.0%	49.0%	100.0%

χ^2=4.019; p=0.045;

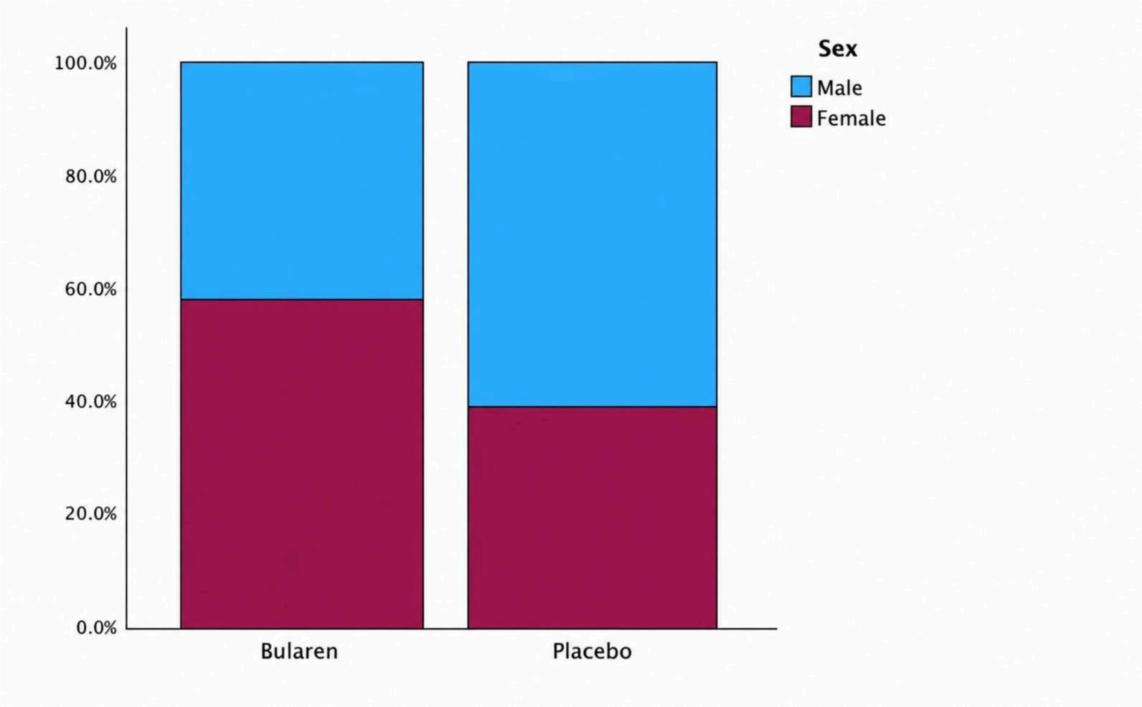

Route of antibiotic administration

			Route of antibiotic administration		
			I.V.	I.V.+PO	All
Treatment	Bularen®	N	5	46	51
		%	9.8%	90.2%	100.0%
	Placebo	N	6	43	49
		%	12.2%	87.8%	100.0%
All		N	11	89	100
		%	11.0%	89.0%	100.0%

χ^2=0.152; p=0.697;

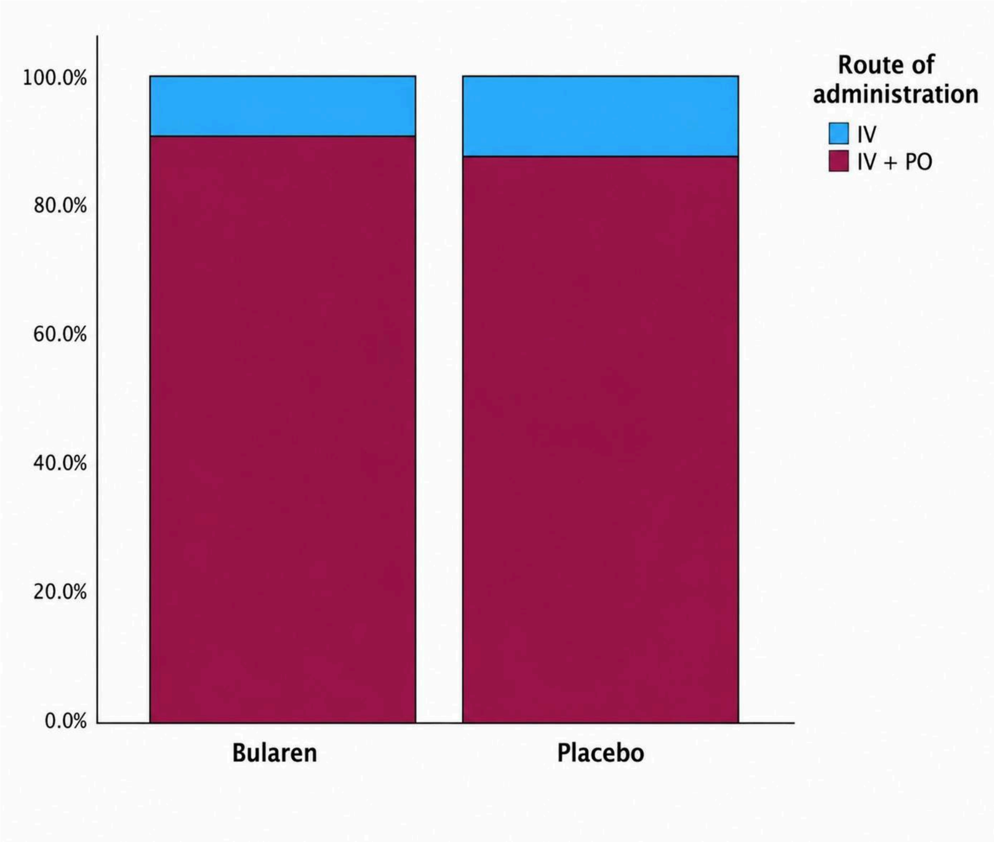

			Br iv Ab.					
			1	2	3	4	5	All
Treatment	Bularen®	N	24	15	10	2	0	51
		%	47.1%	29.4%	19.6%	3.9%	0.0%	100.0%
	Placebo	N	21	20	4	3	1	49
		%	42.9%	40.8%	8.2%	6.1%	2.0%	100.0%
All		N	45	35	14	5	1	100
		%	45.0%	35.0%	14.0%	5.0%	1.0%	100.0%

MW U=1240; Z=-0.071; p=0.944;

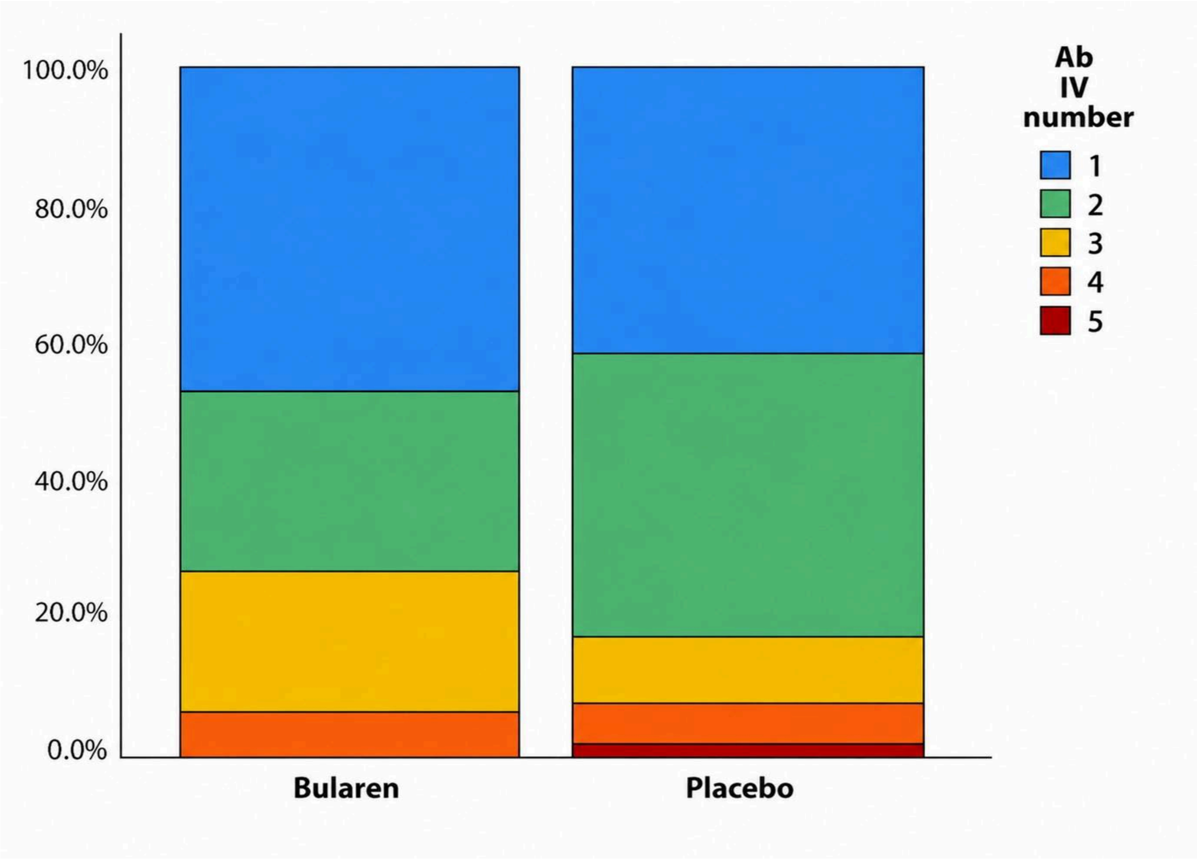

Duration of IV antibiotic therapy, days

		Treatment Bularen®	Placebo
Duration of IV antibiotic therapy, days	N	51	49
	AS	7.1	8.3
	SD	5.6	7.1
	Min	.0	2.0
	P25	4.0	3.0
	Med	6.0	7.0
	P75	8.0	10.0
	Max	28.0	28.0

MW U=1158; Z=-0.634; p=0.526;

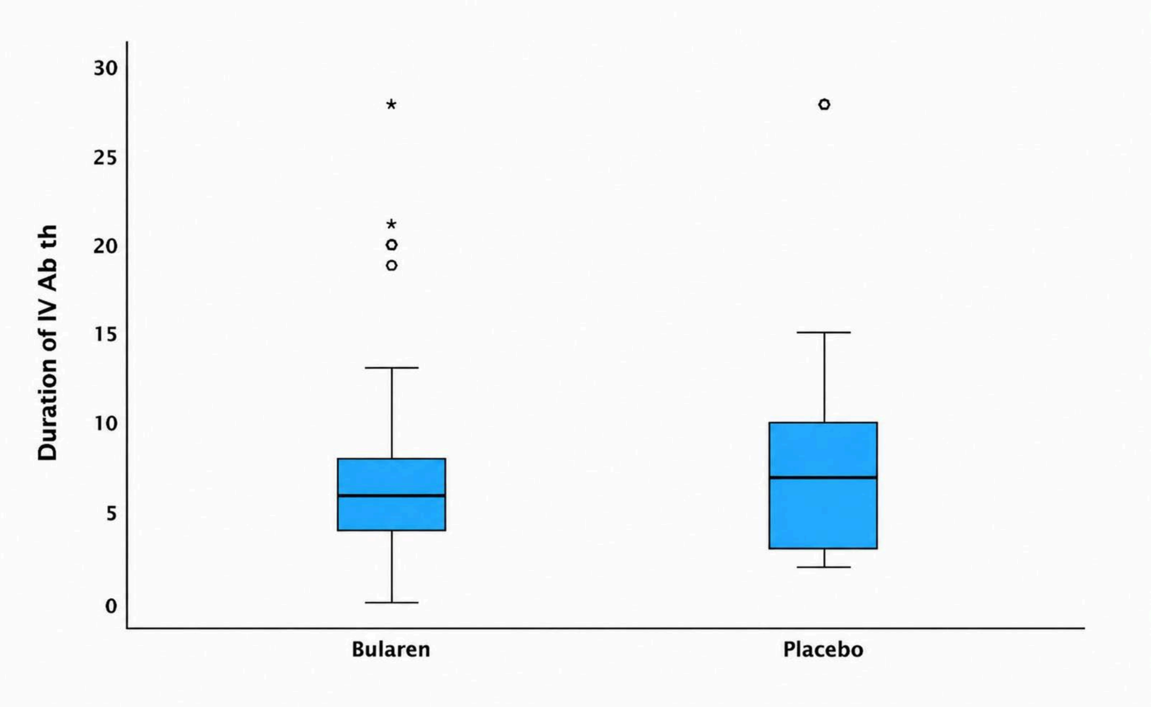

Occurrence of AAD during IV antibiotic therapy

			Occurrence of AAD during IV antibiotic therapy		
			No	Yes	All
Treatment	Bularen®	N	51	0	51
		%	100.0%	0.0%	100.0%
	Placebo	N	36	13	49
		%	73.5%	26.5%	100.0%
All		N	87	13	100
		%	87.0%	13.0%	100.0%

χ^2=15.552; p<0.001;

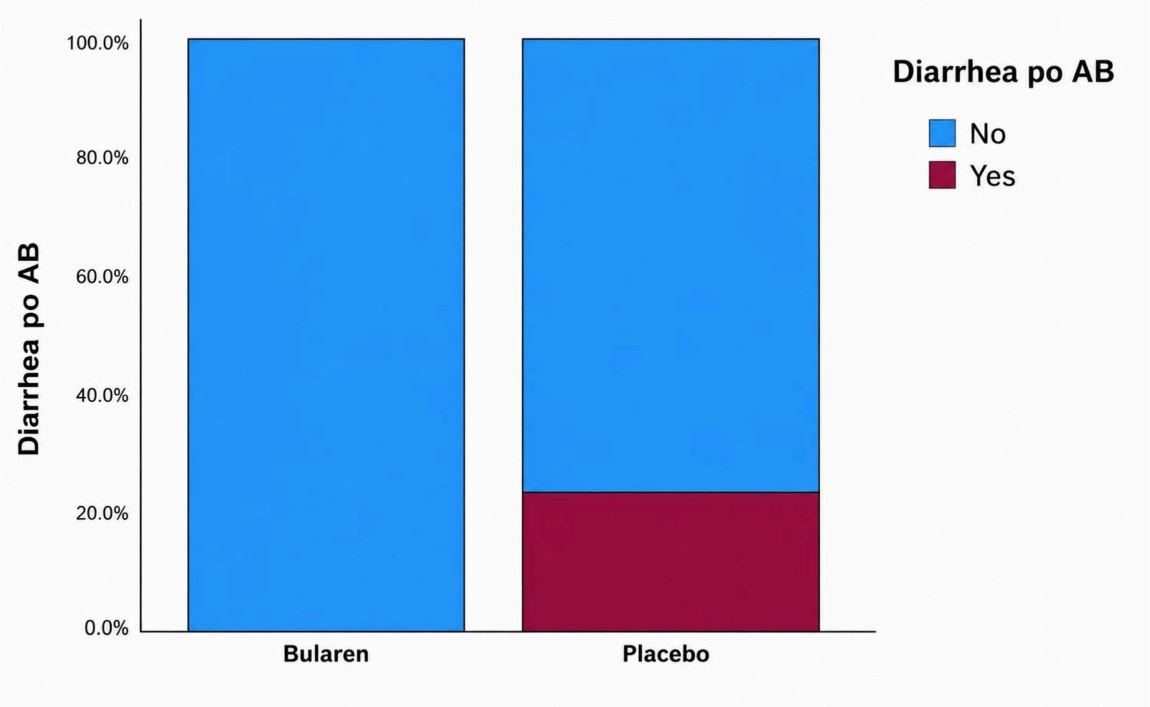

Number of loose or liquid stools per day during IV antibiotic therapy

			Number of loose or liquid stools per day during IV antibiotic therapy	
			2	All
Treatment	Placebo	N	13	13
		%	100.0%	100.0%
All		N	13	13
		%	100.0%	100.0%

Number of loose or liquid stools during IV antibiotic therapy

			Number of loose or liquid stools during IV antibiotic therapy		
			ne	2	All
Treatment	Bularen®	N	51	0	51
		%	100.0%	0.0%	100.0%
	Placebo	N	36	13	49
		%	73.5%	26.5%	100.0%
All		N	87	13	100
		%	87.0%	13.0%	100.0%

Fisher's Exact test p<0.001;

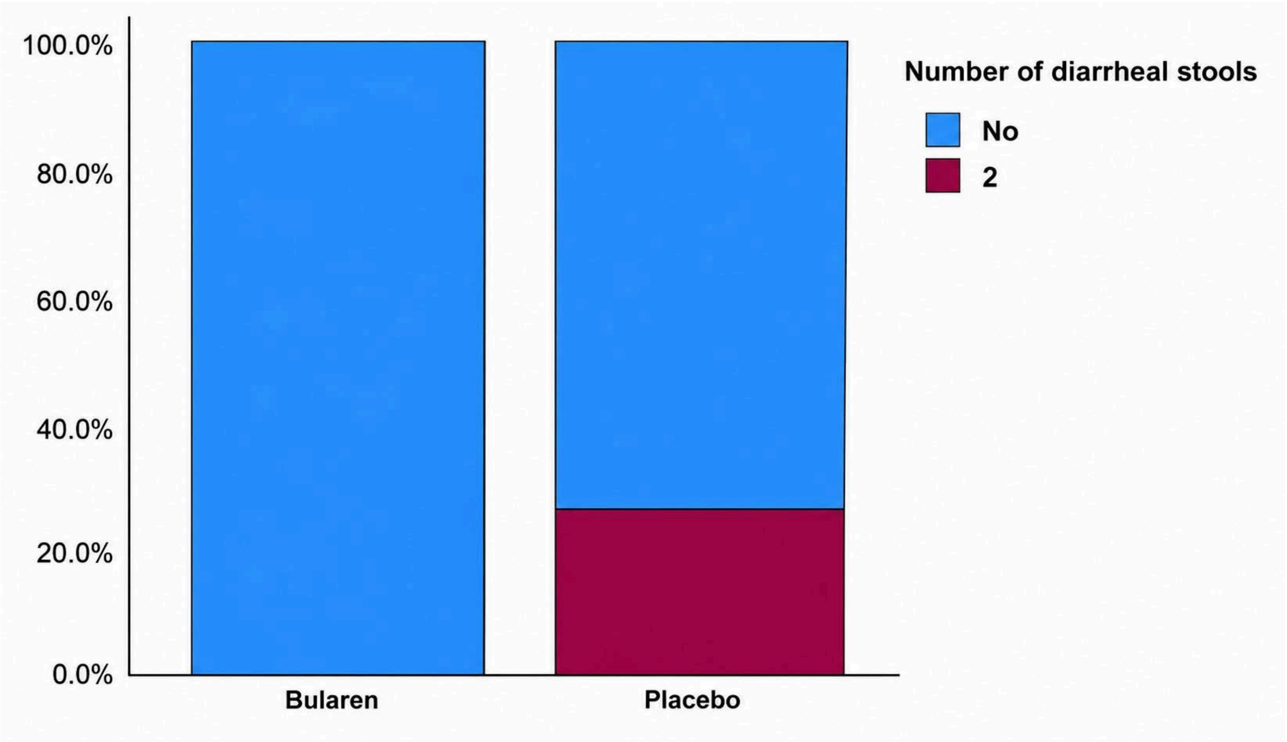

Number of oral antibiotics

			Number of oral antibiotics				
			0	1	2	3	All
Treatment	Bularen®	N	5	38	7	1	51
		%	9.8%	74.5%	13.7%	2.0%	100.0%
	Placebo	N	6	34	7	2	49
		%	12.2%	69.4%	14.3%	4.1%	100.0%
All		N	11	72	14	3	100
		%	11.0%	72.0%	14.0%	3.0%	100.0%

MW U=1241.5; Z=-0.070; p=0.944;

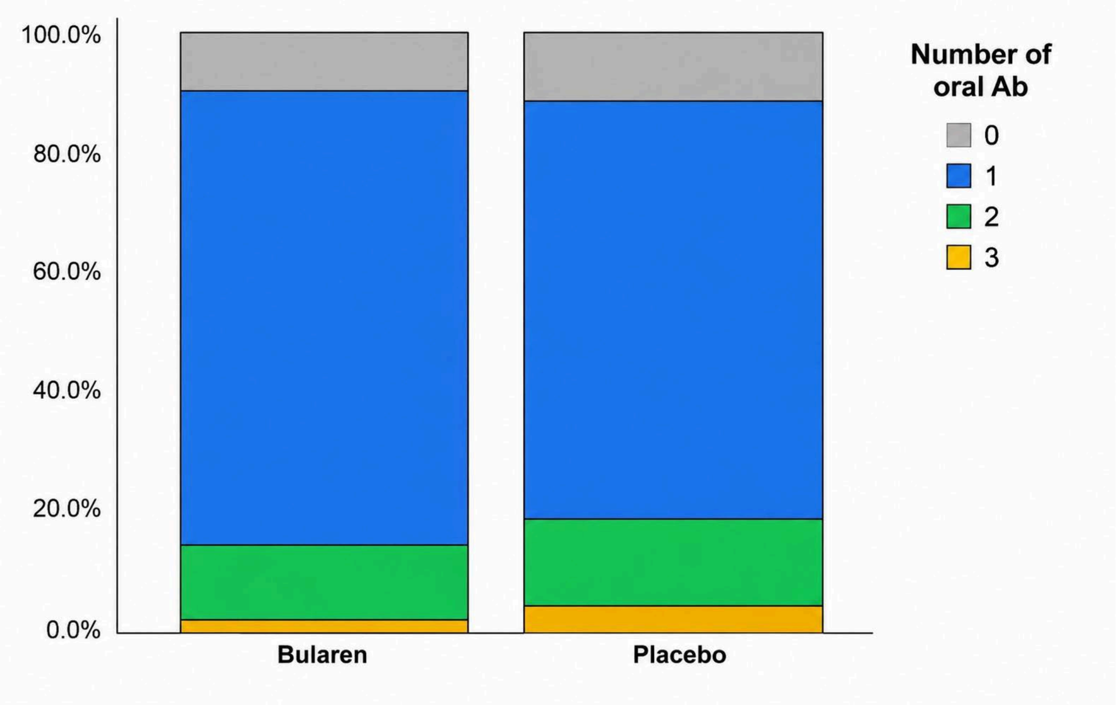

Duration of PO antibiotic therapy, days

		Treatment Bularen®	Placebo
Duration of PO antibiotic therapy, days	N	46	43
	AS	6.3	6.3
	SD	1.7	1.4
	Min	3.0	3.0
	P25	5.0	5.0
	Med	7.0	7.0
	P75	7.0	7.0
	Max	10.0	10.0

MW U=932; Z=-0.512; p=0.609;

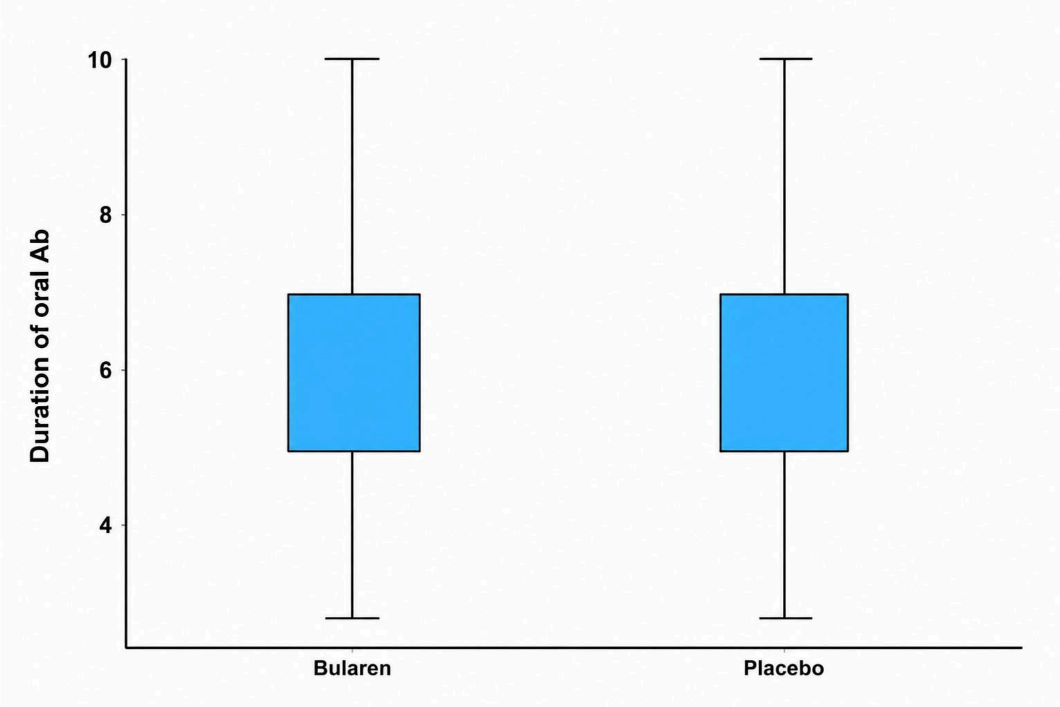

Occurrence of AAD during oral antibiotic therapy

			Occurrence of AAD during oral antibiotic therapy		
			No	Yes	All
Treatment	Bularen®	N	47	4	51
		%	92.2%	7.8%	100.0%
	Placebo	N	24	25	49
		%	49.0%	51.0%	100.0%
All		N	71	29	100
		%	71.0%	29.0%	100.0%

χ^2=22.627; p<0.001;

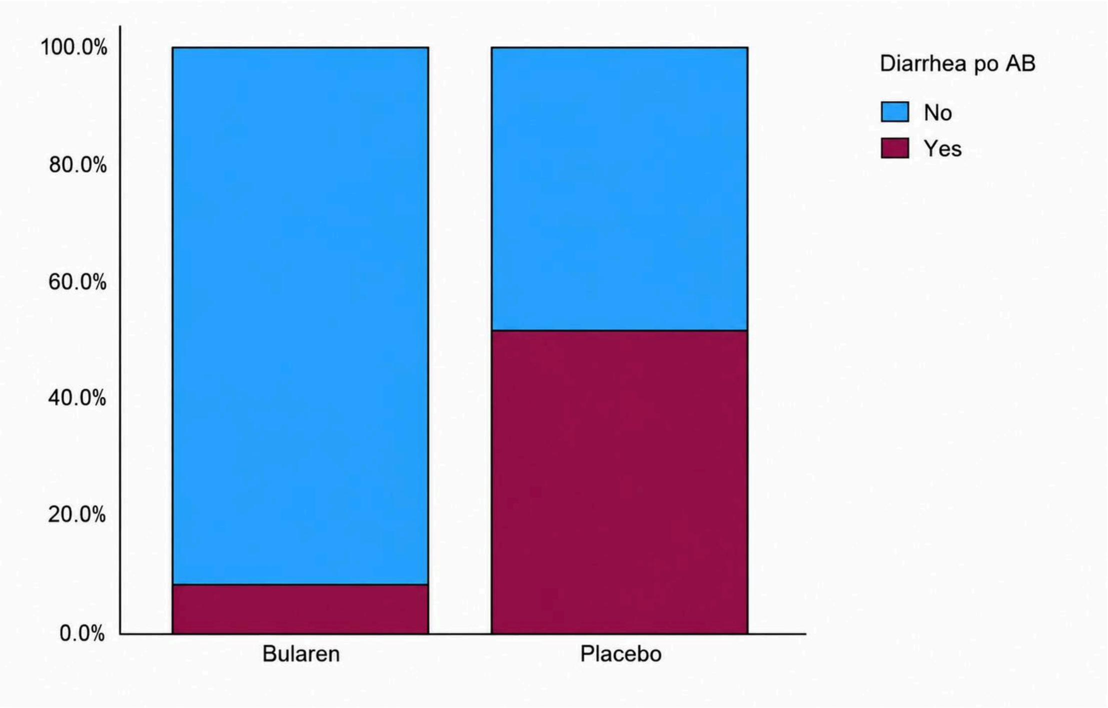

Number of loose or liquid stools per day during oral antibiotic therapy

			Number of loose or liquid stools per day during oral antibiotic therapy		
			2	3	All
Treatment	Bularen®	N	4	0	4
		%	100.0%	0.0%	100.0%
	Placebo	N	21	4	25
		%	84.0%	16.0%	100.0%
All		N	25	4	29
		%	86.2%	13.8%	100.0%

Fisher's Exact test p=1.000;

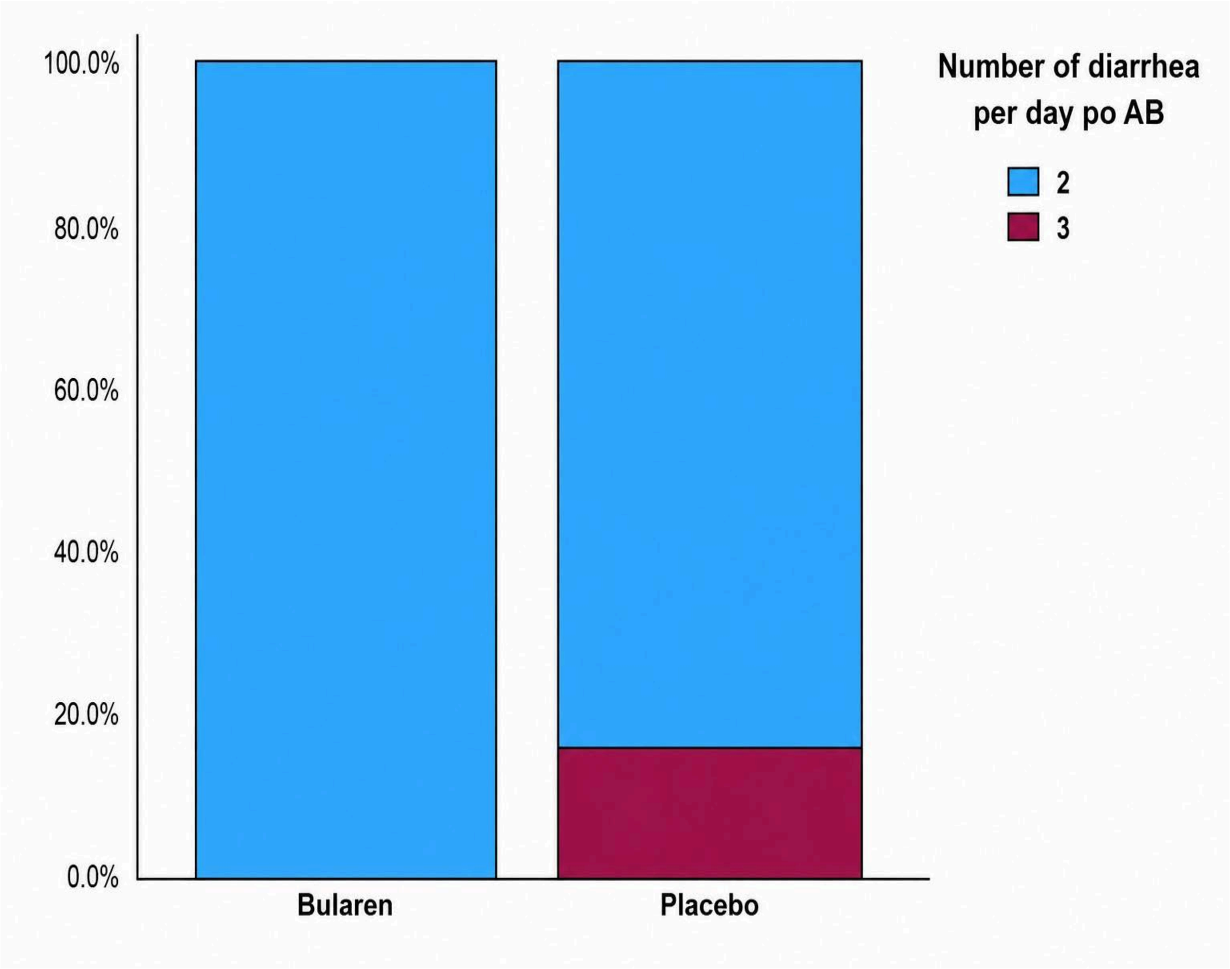

Number of loose or liquid stools during oral antibiotic therapy

			Number of loose or liquid stools during oral antibiotic therapy			
			ne	2	3+	All
Treatment	Bularen®	N	47	4	0	51
		%	92.2%	7.8%	0.0%	100.0%
	Placebo	N	24	21	4	49
		%	49.0%	42.9%	8.2%	100.0%
All		N	71	25	4	100
		%	71.0%	25.0%	4.0%	100.0%

Fisher-Freeman-Halton Exact test $p<0.001$;

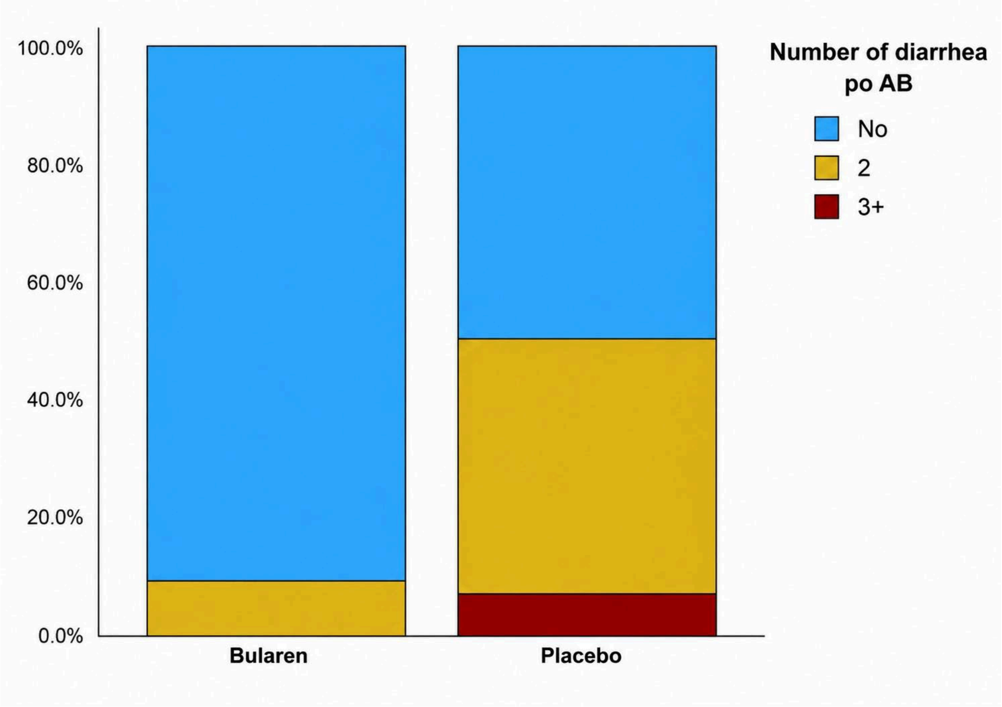

Bloating

			Bloating		
			No	Yes	All
Treatment	Bularen®	N	50	1	51
		%	98.0%	2.0%	100.0%
	Placebo	N	12	37	49
		%	24.5%	75.5%	100.0%
All		N	62	38	100
		%	62.0%	38.0%	100.0%

χ^2=57.379; p<0.001;

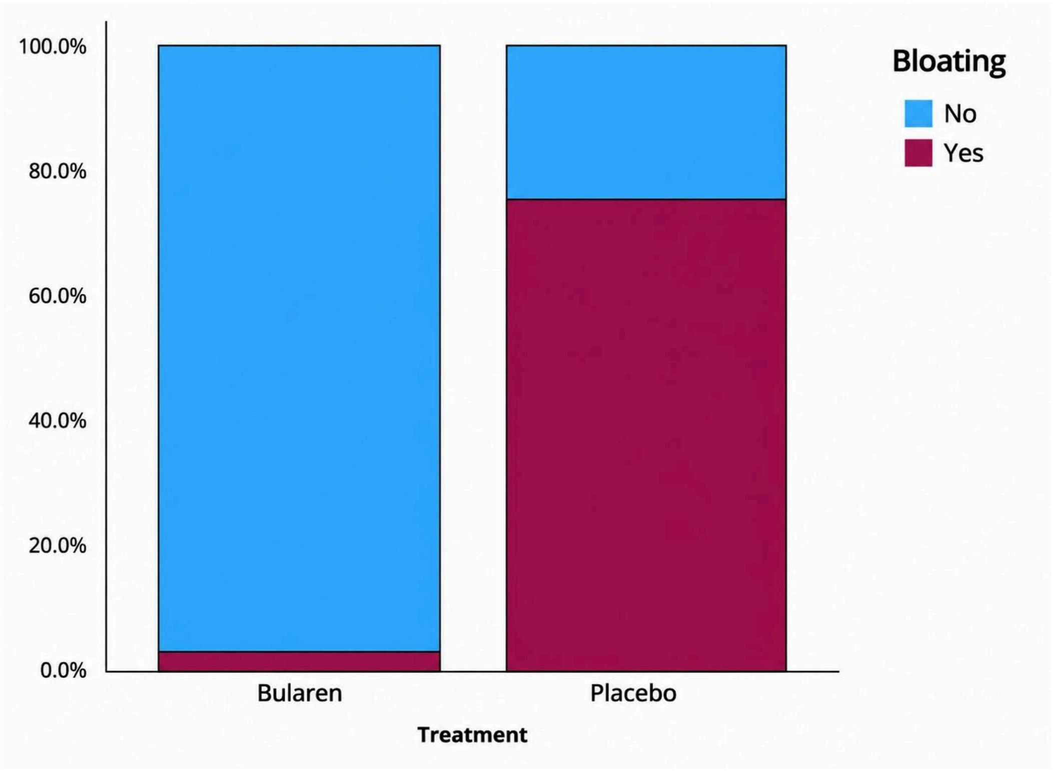

Antibiotics

		Treatment				
		Bularen®		Placebo		*P*
		N	%	N	%	
Amoxicillin/clavulanic acid; oral	No	30	58.8%	35	71.4%	0.186 [a]
	Yes	21	41.2%	14	28.6%	
Piperacillin/tazobactam	No	50	98.0%	49	100.0%	1.000 [b]
	Yes	1	2.0%	0	0.0%	
Ciprofloxacin; oral	No	43	84.3%	37	75.5%	0.271 [a]
	Yes	8	15.7%	12	24.5%	
Moxifloxacin; oral	No	45	88.2%	42	85.7%	0.708 [a]
	Yes	6	11.8%	7	14.3%	
Meropenem	No	41	80.4%	44	89.8%	0.188 [a]
	Yes	10	19.6%	5	10.2%	
Vancomycin	No	48	94.1%	48	98.0%	0.618 [b]
	Yes	3	5.9%	1	2.0%	
Ceftriaxone	No	39	76.5%	31	63.3%	0.150 [a]
	Yes	12	23.5%	18	36.7%	
Metronidazole	No	39	76.5%	37	75.5%	0.910 [a]
	Yes	12	23.5%	12	24.5%	
Cefazolin	No	36	70.6%	36	73.5%	0.748 [a]
	Yes	15	29.4%	13	26.5%	
Gentamicin	No	49	96.1%	48	100.0%	0.495 [b]
	Yes	2	3.9%	0	0.0%	
Amoxicillin/clavulanic acid	No	37	72.5%	37	75.5%	0.736 [a]
	Yes	14	27.5%	12	24.5%	
Ciprofloxacin	No	38	74.5%	34	69.4%	0.568 [a]
	Yes	13	25.5%	15	30.6%	
Cefixime	No	45	88.2%	36	73.5%	0.060 [a]
	Yes	6	11.8%	13	26.5%	
Doxycycline	No	49	96.1%	47	95.9%	1.000 [b]
	Yes	2	3.9%	2	4.1%	
Clindamycin	No	46	90.2%	47	95.9%	0.437 [b]
	Yes	5	9.8%	2	4.1%	
Azithromycin; oral	No	45	88.2%	48	98.0%	0.112 [b]
	Yes	6	11.8%	1	2.0%	
Metronidazole; oral	No	45	88.2%	45	91.8%	0.741 [b]
	Yes	6	11.8%	4	8.2%	
Azithromycin	No	48	94.1%	47	95.9%	1.000 [b]
	Yes	3	5.9%	2	4.1%	
Moxifloxacin	No	48	94.1%	46	93.9%	1.000 [b]
	Yes	3	5.9%	3	6.1%	
Cefepime	No	50	98.0%	46	93.9%	0.357 [b]
	Yes	1	2.0%	3	6.1%	
Trimethoprim–sulfamethoxazole	No	51	100.0%	48	98.0%	0.490 [b]
	Yes	0	0.0%	1	2.0%	
Amikacin	No	51	100.0%	47	95.9%	0.238 [b]
	Yes	0	0.0%	2	4.1%	
Colistin	No	51	100.0%	48	98.0%	0.490 [b]
	Yes	0	0.0%	1	2.0%	
Ceftazidime + avibactam	No	51	100.0%	48	98.0%	0.490 [b]
	Yes	0	0.0%	1	2.0%	

[a]Pearson chi square test [b]Fisher's Exact test
